# Ex-vivo right posterior sectionectomy for large-for-size mismatch in pediatric liver transplant recipients

**DOI:** 10.1007/s00423-026-04320-y

**Published:** 2026-10-13

**Authors:** Andrea Della Penna, Steffen Hartleif, Markus Quante, Silvio Nadalin

**Affiliations:** 1 Department of General, Visceral and Transplant Surgery, University Hospital Tübingen, Hoppe-Seyler-Straße 3, 72076 Tübingen, Germany; 2 Paediatric Gastroenterology and Hepatology, Department of Haematology and Oncology, University Children’s Hospital Tübingen, Tübingen, Germany; 3 Department of General, Visceral and Transplant Surgery, Hannover Medical School, Hannover, Germany

**Keywords:** Liver transplantation, Pediatric transplantation, Graft reduction, Surgical techniques, Large-for-size syndrome

## Abstract

**Purpose:**

Large-for-size mismatch remains a major challenge in pediatric and small recipient liver transplantation, particularly when full-size deceased-donor grafts are allocated and conventional splitting is not feasible.

**Methods:**

We performed a single-center retrospective analysis of seven pediatric and adolescent recipients who underwent deceased donor liver transplantation (DDLT) with ex-vivo right posterior sectionectomy (RPS) between 2018 and 2022. Grafts were considered oversized based on an integrated surgical assessment including graft-to-recipient weight ratio (GRWR), recipient abdominal anatomy, graft morphology, and expected graft fit.

**Results:**

Median GRWR decreased from 4.7% to 2.2%, allowing graft implantation in all cases. Primary abdominal closure was achieved in six recipients, while one required delayed closure for large-for-size syndrome (LFS). No cases of severe LFS with hemodynamic, ventilatory, or graft perfusion compromise were observed. Major complications occurred in three patients, including one biliary complication requiring surgical revision, one primary graft failure requiring retransplantation, and one death unrelated to graft size or transplant surgery. At a median follow-up of 59.5 months among surviving recipients, six of seven patients were alive; five retained their original graft, while one was alive with a functioning second graft after retransplantation.

**Conclusion:**

Ex-vivo RPS appears to be a technically feasible rescue graft reduction strategy in highly selected pediatric recipients when conventional graft reduction techniques are not feasible. Given the limited cohort size and the occurrence of primary graft failure and mortality, its safety and efficacy require further evaluation.

## Introduction

Large-for-size syndrome (LFS) is a widely recognized complication in pediatric and small adult liver transplantation recipients, resulting from a mismatch between graft volume and recipient abdominal capacity. It leads to graft compression, impaired inflow and outflow, increased intra-abdominal pressure, and, in severe cases, the inability to achieve primary abdominal closure [1, 2]. LFS has both mechanical and hemodynamic components, with compression of hepatic parenchyma and increased portal inflow leading to early graft dysfunction. Although definitions have varied, graft-to-recipient weight ratio (GRWR) values above 4% in pediatric recipients have been consistently associated with increased risk of LFS and abdominal compartment syndrome [1–4]. Although no universally accepted GRWR threshold exists, values below approximately 3% are generally considered desirable whenever technically feasible to reduce the risk of LFS. However, LFS cannot be explained solely by graft volume or GRWR. Instead, it reflects a three-dimensional mismatch between graft morphology and the recipient abdominal domain, in which the spatial relationship between graft shape and available abdominal space plays a critical role [1, 2]. Importantly, graft oversizing may occur along different spatial directions. While mismatch may predominate in a latero–lateral or cranio–caudal axis, particularly in full-size liver grafts it frequently manifests in the anteroposterior direction, reflecting a mismatch between graft thickness and the limited depth of the recipient abdominal cavity [5, 6].

Several surgical strategies have been developed to overcome graft–recipient size mismatch, including reduced-size, split-liver, and living donor liver transplantation [7–9]. These approaches have significantly expanded the pediatric donor pool but may not always be feasible, particularly in urgent situations or when donor or graft characteristics preclude conventional splitting. Recent reviews have emphasized the technical refinement of bench reductions and their role in pediatric transplant practice [10]. In full-size liver grafts, the anteroposterior mismatch predominantly involves the right subdiaphragmatic compartment, where the available space is anatomically constrained by the lower right hemithorax and rib cage, resulting in limited expandability. As a consequence, even grafts with apparently acceptable GRWR values may cause graft compression and venous outflow impairment due to excessive anteroposterior graft thickness rather than overall graft weight [5, 6].

Ex-vivo right posterior sectionectomy (RPS) has recently emerged as a rational reduction strategy when conventional split is not feasible, because it preserves the right hepatic vein and most of the right anterior sector, ensuring adequate functional volume while achieving a significant decrease in graft size [5, 11]. By selectively reducing the posterior mass of the right lobe, ex-vivo RPS specifically addresses the anteroposterior component of graft–recipient mismatch, which is particularly relevant when whole liver grafts are used in recipients with limited right subdiaphragmatic abdominal depth [5, 12]. The HuaXi group first described this technique in 5 adults, demonstrating feasibility and favorable early outcomes [5]. Since then, only a few reports have been published, including a small adolescent series [12] and technical notes in adult recipients [6, 11, 13]. Evidence in pediatric and small adult recipients remains extremely limited. In this context, alternative strategies such as right hepatectomy, left lateral segment grafts, or left hepatectomy may be considered; however, these approaches are not always feasible due to donor-related factors, graft injury, or the need to preserve sufficient functional volume, particularly in urgent settings.

In this study, we report our single-center experience with ex-vivo RPS as a rescue graft reduction strategy for highly selected pediatric and adolescent recipients with oversized whole liver grafts when conventional graft splitting was not feasible, most often under high-urgency conditions. Our aim was to evaluate the technical feasibility of the procedure and the associated clinical outcomes of this tailored approach in deceased donor liver transplantation (DDLT).

## Methods

### Study design and patient population

We conducted a retrospective, single-center analysis of recipients who underwent DDLT with graft reduction by ex-vivo RPS at the Department of General, Visceral, and Transplant Surgery, University Hospital Tübingen, between January 2018 and April 2022. This retrospective study included all consecutive pediatric and adolescent recipients who underwent ex-vivo RPS at our institution during the study period. Eligible recipients were children and adolescents in whom the allocated whole liver graft was considered oversized on the basis of an integrated assessment of GRWR, recipient abdominal anatomy, graft morphology, apparent anteroposterior thickness, and the expected fit of the graft within the right subdiaphragmatic space. A pre-reduction GRWR above approximately 4% was present in most, but not all, recipients and was not used as an isolated indication for RPS. Where available, pretransplant CT imaging was reviewed to assess the recipient abdominal domain and right upper quadrant anatomy. The indication for ex-vivo RPS was not based on a single predefined morphometric threshold but on an integrated surgical assessment combining recipient size, pre-reduction GRWR, recipient abdominal anatomy on preoperative imaging, graft morphology, and the feasibility of conventional graft splitting. Particular attention was given to the apparent anteroposterior thickness of the graft during back-table evaluation and its expected fit within the recipient’s right subdiaphragmatic space. Accordingly, in addition to volumetric considerations, whole liver grafts were considered oversized when their morphology and apparent thickness were deemed incompatible with the recipient right subdiaphragmatic space during back-table assessment, prompting graft reduction by ex-vivo RPS. Whenever feasible, standard graft reduction strategies were preferred. Ex-vivo RPS was therefore reserved for highly selected rescue situations in which conventional split-liver transplantation was not feasible and preservation of adequate functional graft volume was considered essential. In individual cases, graft-specific findings, such as traumatic injury involving the posterior sector, also contributed to the decision-making process. The grafts were accepted via the Eurotransplant allocation process but were subsequently deemed unsuitable for conventional splitting according to Eurotransplant criteria, or presented parenchymal lesions that precluded safe splitting. In these latter cases, ex-vivo RPS was performed as a rescue bench procedure to keep the graft transplantable. Indications for transplantation included acute liver failure, hepatoblastoma, and chronic cholestatic liver disease.

### Donor and graft characteristics

Donor and graft data, including age, sex, body mass index (BMI), cause of death, and Eurotransplant donor risk index (ET-DRI), were collected from institutional transplant records. The GRWR was calculated as graft weight (g) divided by recipient weight (g) × 100 and is reported as a percentage. The degree of graft reduction was determined by comparing graft weight before and after RPS and was expressed as both absolute graft weight reduction (g) and percentage of graft weight removed.

### Surgical technique

**Fig. 1:**
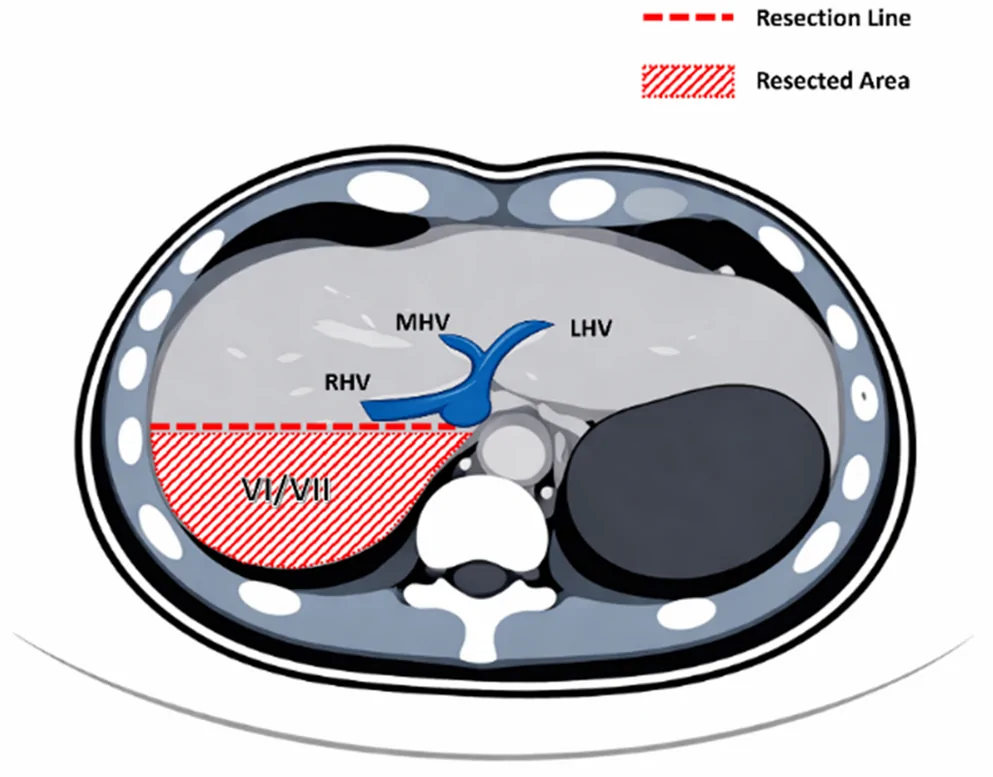
Conceptual illustration of ex-vivo right posterior sectionectomy (RPS). Axial schematic representation of the liver demonstrating the relationship between the hepatic veins and the planned resection plane. The right hepatic vein (RHV) defines the transection plane separating the posterior section (segments VI–VII, shaded) from the remaining graft. Ex-vivo RPS allows reduction of graft volume while preserving venous outflow of the anterior sector

**Fig. 2:**
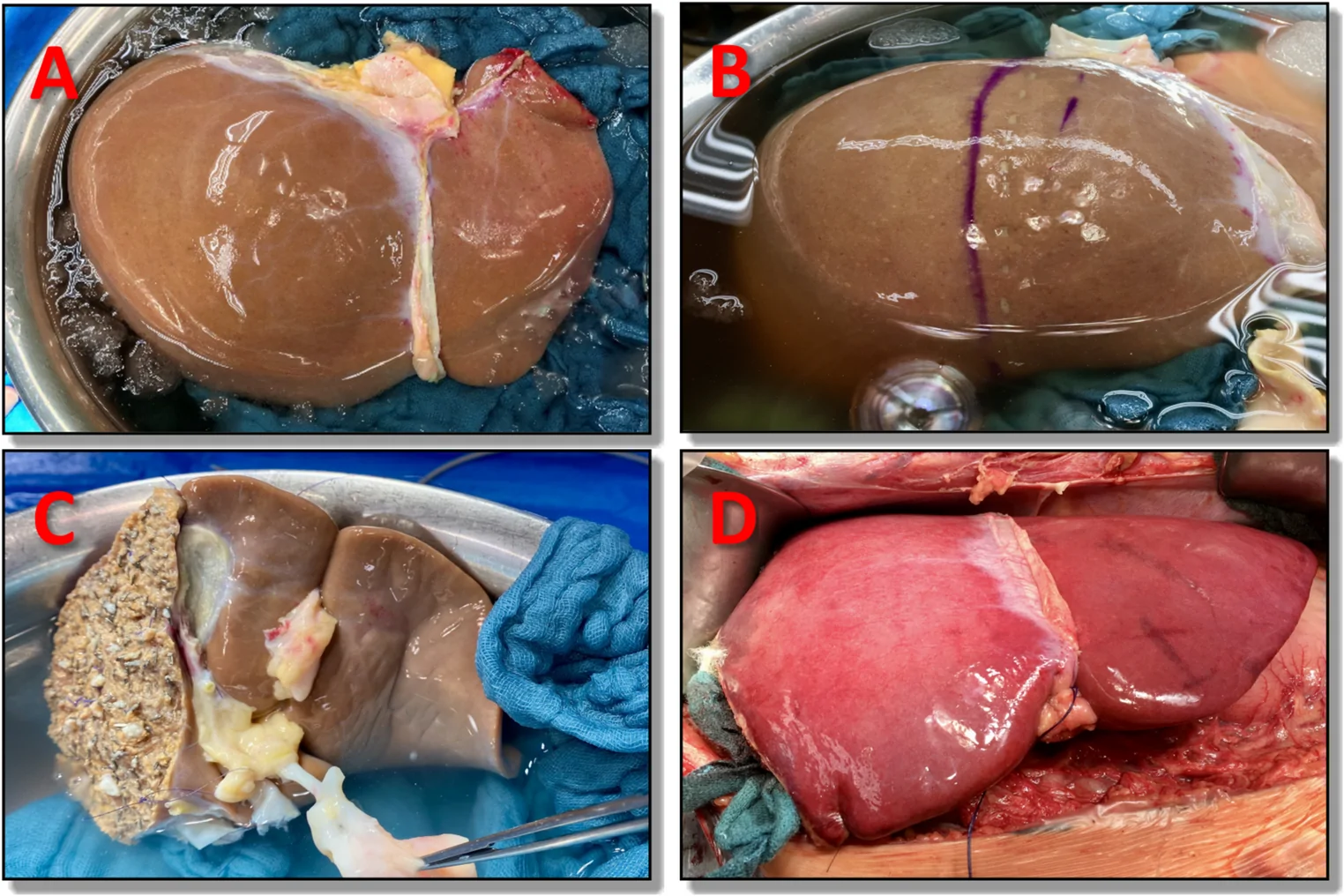
Representative procedural steps of ex-vivo right posterior sectionectomy (RPS**).** (**A**) Whole liver graft before reduction. (**B**) Planned transection line marked on the liver surface. (**C**) Reduced graft after ex-vivo right posterior sectionectomy demonstrating the final reduced graft. (**D**) Final appearance of the reduced graft after implantation and reperfusion

All graft reductions were performed ex-vivo on the back table under static cold preservation. After removal from the transport bag, the graft was placed in a basin containing ice and cold saline solution and flushed through the portal vein with cold histidine–tryptophan–ketoglutarate (HTK) solution before back-table preparation and graft reduction. Cold preservation was maintained throughout the entire bench procedure. Machine perfusion was not used in any case during the study period. Similar to the HuaXi technique [5, 11], the transection plane was delineated along the course of the right hepatic vein, which was carefully preserved throughout the procedure. The right hepatic vein was identified by blunt dissection using forceps, and its course was then palpatorily traced and marked on the liver surface to guide parenchymal transection. The transection plane was defined along the right hepatic vein, enabling removal of the posterior section (segments VI–VII) while preserving venous outflow of the anterior sector (Fig. 1). The parenchymal transection was performed using a Cavitron Ultrasonic Surgical Aspirator (CUSA; Integra LifeSciences, Princeton, NJ, USA), allowing controlled dissection and identification of intrahepatic vascular and biliary structures. The Glissonean pedicles to segments VI and VII were identified, ligated, and divided during transection. Hemostasis and biliary integrity were carefully assessed throughout parenchymal transection, with final inspection of the resection surface performed before implantation. The key steps of the reduction procedure are illustrated in Fig. 2.

### Outcomes and definitions

The primary outcome was the incidence of LFS. Given the heterogeneous definitions of LFS reported in the literature [1, 2], for the purposes of this study, LFS was defined clinically as graft–recipient size mismatch preventing safe primary fascial closure and requiring temporary or staged abdominal closure. Severe LFS was defined by additional hemodynamic, ventilatory, or graft perfusion compromise attributable to abdominal graft compression. Secondary outcomes included delayed abdominal closure, perioperative morbidity graded according to the Clavien–Dindo classification, early vascular and biliary complications, graft and patient survival, and long-term graft function. Outcomes were evaluated up to the last available follow-up for each patient. Reduction time was defined as the interval from initiation of back-table graft preparation until completion of the bench procedure, when the reduced graft was fully prepared and ready for implantation. This interval was entirely included in the reported cold ischemia time and reflects the complete back-table workflow rather than the duration of parenchymal transection alone.

### Statistical analysis

Data were analyzed using descriptive statistics. Continuous variables are presented as median and range, and categorical variables as counts and percentages. No comparative statistical testing was performed given the limited sample size. Statistical analysis was performed using SPSS Statistics (IBM Corp., Armonk, NY, USA).

### Ethics statement

This study was a retrospective analysis of pseudonymized routinely collected clinical data conducted as institutional self-research at the Department of General, Visceral and Transplant Surgery, University Hospital Tübingen. According to § 13 of the Baden-Württemberg State Data Protection Act (Landesdatenschutzgesetz Baden-Württemberg), retrospective analyses conducted as institutional self-research using existing pseudonymized clinical data do not require formal approval by an ethics committee. No additional informed consent was obtained because the analysis was restricted to existing pseudonymized clinical data and was conducted under the applicable provisions for institutional research. The study was conducted in accordance with the Declaration of Helsinki.

## Results

### Recipient characteristics

During the study period, 78 pediatric liver transplantations were performed at our institution, including 55 DDLT. Only seven recipients (five females and two males; median age 11 years, range 2–18) underwent DDLT with ex-vivo RPS and constitute the present study cohort (Table 1). Indications for transplantation included acute liver failure in four patients, oncologic disease (hepatoblastoma) in two patients, and chronic cholestatic liver disease due to PFIC (MDR3 deficiency) in one patient (Table 1). Six recipients were listed under high-urgency (HU) status, including all four patients with acute liver failure and two patients with hepatoblastoma who met Eurotransplant criteria for HU listing based on their underlying disease and clinical condition. Three recipients were admitted to the intensive care unit prior to transplantation (Table 1).

**Table 1:** Recipient characteristics

Patient	Age (y)	Sex	Weight (kg)	Height (cm)	BMI	High urgency	Indication for liver transplantation	ICU pre-transplant	Waiting time (days)
1	13	F	46.8	153	20.0	Yes	ALF – Wilson disease	No	7
2	18	F	73.8	163	27.8	No	Chronic cholestatic liver disease – PFIC (MDR3 deficiency)	No	883
3	11	F	36.0	140	18.4	Yes	ALF – unclear etiology	Yes	1
4	2	F	11.0	90	13.6	Yes	Hepatoblastoma	No	13
5	3	M	11.0	89	13.8	Yes	Hepatoblastoma	No	4
6	15	F	60.0	160	23.4	Yes	ALF – paracetamol intoxication	Yes	2
7	7	F	32.0	120	22.2	Yes	ALF – autoimmune hepatitis	Yes	3

*ALF* acute liver failure, *BMI* body mass index, *ICU* intensive care unit, *PFIC* progressive familial intrahepatic cholestasis. High urgency indicates listing under Eurotransplant high-urgency criteria at the time of transplantation

### Donor and graft characteristics

**Fig. 3:**
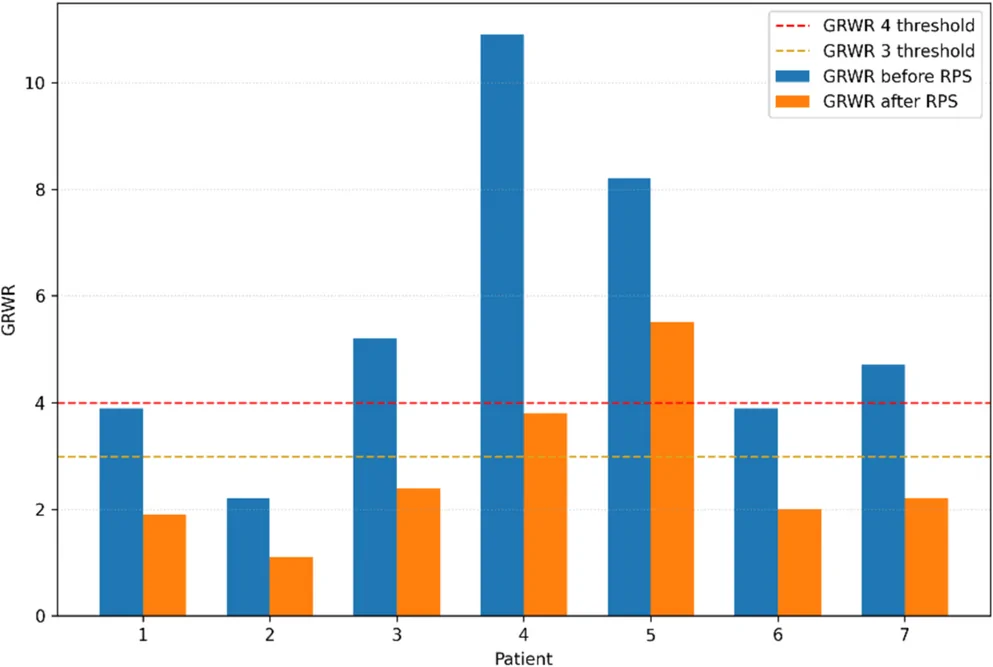
Change in graft-to-recipient weight ratio before and after ex-vivo right posterior sectionectomy. Bar charts illustrate graft-to-recipient weight ratio (GRWR) for each recipient before graft reduction (whole graft) and after ex-vivo right posterior sectionectomy. Horizontal dashed lines indicate commonly used GRWR thresholds of 4% and 3%. Ex-vivo graft reduction resulted in a consistent decrease in GRWR across all cases

Median donor age was 22 years (range 8–61) (Table 2). All grafts were whole livers judged unsuitable for splitting according to Eurotransplant splitting criteria or presented parenchymal injuries that rendered splitting unsafe (Table 3). In these cases, RPS was performed as a rescue bench procedure allowing utilization of grafts otherwise unsuitable for the intended small recipients. The median GRWR decreased from 4.7% (range 2.2–10.9) before reduction to 2.2% (range 1.1–5.5) after RPS; in five of seven patients, the final GRWR was below 3% (Table 2; Fig. 3). The corresponding graft weights before and after reduction, together with the percentage of graft weight removed, are summarized in Table 2. Median cold ischemia time was 8 h 37 min (range 7 h 04 min–10 h 20 min), and median warm ischemia time was 26 min (range 23–31 min). Median graft reduction time was 121 min (range 38–158 min).

**Table 2:** Donor and graft characteristics

Patient	Donor age (y)	Donor sex	Donor weight (kg)	Donor BMI	Cause of death	Whole liver weight (g)	Resected tissue (g)	Final graft weight (g)	Graft weight reduction (%)	GRWR, whole graft (%)	GRWR, after RPS (%)	ET-DRI	DRI
1	22	M	80	24.0	Trauma	1812	895	917	49.4	3.9	1.9	1.17	1.99
2	16	F	80	23.0	Trauma	1607	804	803	50.0	2.2	1.1	1.20	2.05
3	43	M	90	25.0	Anoxia	1859	975	884	52.4	5.2	2.4	1.89	2.84
4	20	F	70	21.0	Trauma	1200	781	419	65.1	10.9	3.8	1.09	1.64
5	8	M	36	15.0	Trauma	900	300	600	33.3	8.2	5.5	1.50	2.30
6	61	F	65	22.0	Stroke	2393	1055	1238	44.1	3.9	2.0	1.40	2.00
7	19	M	72	23.0	Trauma	1526	810	716	53.1	4.7	2.2	1.25	2.10

*BMI* body mass index, *DRI* donor risk index, *ET-DRI* Eurotransplant donor risk index, *GRWR* graft-to-recipient weight ratio, *RPS* right posterior sectionectomy. GRWR values are reported before and after graft reduction by ex-vivo right posterior sectionectomy

**Table 3:** Eurotransplant splittability assessment and reasons for graft non-splittability

Donor	Eurotransplant criteria met	Eurotransplant reason for non-splittability	Technical or clinical reason for non-splittability
1	No	Cardiac arrest, 15‘ cardiopulmonary resuscitation; anoxic injury	—
2	Yes	—	Grade 3 parenchymal injury
3	No	Cardiac arrest, 40‘ cardiopulmonary resuscitation	—
4	No	Cold ischemia time > 10 h	—
5	No	Donor age 8 years; donor weight 36 kg (< 50 kg)	—
6	No	Intensive care unit stay > 5 days	—
7	No	Donor age 61 years; ICU stay > 5 days	—

Eurotransplant criteria refer to standard allocation and splitting guidelines applied at the time of organ offer. Technical or clinical reasons indicate additional graft-related findings identified during surgical assessment that precluded safe graft splitting

### Postoperative outcomes

Primary abdominal closure was achieved in six patients (Table 4). In patient 5, persistent graft–recipient size mismatch despite ex-vivo RPS (final GRWR 5.5%) precluded safe primary fascial closure, fulfilling the study definition of LFS. This patient was a very small recipient (11 kg) transplanted for hepatoblastoma in the presence of an Abernethy type II malformation. Temporary abdominal closure using an expanded polytetrafluoroethylene (ePTFE) patch was therefore performed, followed by definitive fascial closure on postoperative day 4. No cases of severe LFS with hemodynamic, ventilatory, or graft perfusion compromise were observed. Major complications (Clavien–Dindo ≥ III) occurred in three patients (Table 4), including biliary anastomotic insufficiency requiring surgical revision with takedown of the duct-to-duct anastomosis and conversion to a biliodigestive anastomosis (grade IIIb), primary graft failure (grade IVb), and death (grade V). No vascular complications or biliary strictures were observed among the six surviving patients.

**Table 4:** Postoperative outcomes and follow-up

Patient	Primary abdominal closure	Highest Clavien–Dindo grade	Main complications	Retransplantation	Death	Outcome at last follow-up	Follow-up (months)
1	Yes	0	—	No	No	Alive with functioning graft	82
2	Yes	II	Anemia; renal insufficiency	No	No	Alive with functioning graft	76
3	Yes	V	Death due to cerebral edema (POD 34)	No	Yes	Deceased	—
4	Yes	I	Oliguria	No	No	Alive with functioning graft	65
5	No	IIIb	Biliary anastomotic insufficiency; open abdomen	No	No	Alive with functioning graft	54
6	Yes	IVb	Primary graft failure	Yes	No	Alive with functioning second graft	43
7	Yes	II	Abdominal collections	No	No	Alive with functioning graft	36

*POD* postoperative day, *FU* follow-up. Clavien–Dindo grade refers to the highest postoperative complication severity per patient. Primary closure refers to abdominal wall closure at index transplantation. Large-for-size syndrome (LFS) was diagnosed in Patient 5, requiring delayed fascial closure

### Survival and long-term outcomes

At last follow-up, patient survival was 6/7 (85.7%). One patient (1/7, 14.3%) underwent retransplantation because of primary graft failure. Of the six surviving recipients, five retained their original graft, while the retransplanted patient was alive with a functioning second graft. Median follow-up among surviving recipients was 59.5 months (range 36–82 months) (Table 4).

The 11-year-old patient who died on postoperative day (POD) 34 had presented with fulminant acute liver failure of unknown etiology and severe intracranial hypertension prior to transplantation. Despite emergent liver transplantation and decompressive craniectomy, the patient suffered extensive herniation-related ischemic brain injury with persistent coma and tetraplegia. In view of the irreversible neurological damage, care was eventually limited to best supportive care, and the patient finally died due to progressive multi-organ failure. No liver transplant–related surgical complications or features of LFS were observed in this patient.

The 15-year-old patient requiring retransplantation initially developed primary graft failure of the reduced-size graft, with histopathological examination demonstrating extensive ischemic necrosis. The patient underwent retransplantation on POD 22 with a full-size liver graft. The second donor graft originated from a smaller donor and was considered more appropriate for the recipient abdominal cavity, allowing implantation without further graft reduction. Early after the second transplant, the patient developed biopsy-proven early vascular rejection, which responded to high-dose methylprednisolone. At the last follow-up, 43 months after retransplantation, she is alive with good graft function (Table 4).

## Discussion

In pediatric and small adult recipients with urgent indications for liver transplantation, LFS continues to represent a critical challenge. This study describes our experience with ex-vivo RPS as a rescue graft reduction strategy in a highly selected cohort of pediatric and adolescent recipients. Notably, the vast majority of patients in this series were transplanted under high-urgency conditions, often for acute liver failure, where waiting for a more appropriate graft was not feasible. Accordingly, our findings should not be extrapolated to routine pediatric liver transplantation and should instead be interpreted within the context of urgent rescue situations in which conventional graft-matching strategies are not feasible.

In our cohort, alternative graft reduction strategies such as right hepatectomy, left lateral segment grafts, or left hepatectomy were not considered suitable, either due to donor-related factors, graft injuries, or the risk of insufficient functional graft volume. An important limitation of our study is that the indication for ex-vivo RPS was based on an integrated surgical assessment rather than on predefined quantitative morphometric criteria. During the study period, no validated measurements of graft thickness or three-dimensional graft-recipient mismatch were available to guide decision-making. Consequently, patient selection relied on the combination of GRWR, recipient abdominal anatomy, graft morphology, expected graft fit, and intraoperative surgical judgement, with particular emphasis on the anteroposterior graft dimension and its accommodation within the right subdiaphragmatic space. Future studies incorporating standardized CT-based morphometric analyses may help establish more objective selection criteria for this technique. In this setting, ex-vivo RPS represented a tailored surgical compromise, allowing adequate graft downsizing while preserving venous outflow and sufficient functional liver mass.

Previous studies have emphasized the importance of optimal graft sizing in liver transplantation, highlighting GRWR and graft morphology as important determinants of outcome [3, 14]. Traditional strategies, such as split-liver transplantation, reduced-size grafts, and living donor transplantation, have improved graft–recipient matching but are not universally applicable, especially in emergency settings or when donor anatomy or graft injury precludes splitting. In these situations, bench graft reduction may represent an important alternative, as emphasized by Kasahara and colleagues [10]. Within this spectrum, ex-vivo RPS represents a precise and anatomically targeted form of graft reduction that maintains venous outflow through preservation of the right hepatic vein while effectively decreasing graft volume [5, 11].

Published clinical experience with right posterior sectionectomy for graft reduction remains extremely limited (Table 5). Following the pilot series by Pu et al., published experience has remained limited to a small pediatric case series by Rossignol et al., a technical report of in situ right posterior sectionectomy by Zhang et al., and a four-patient adult series recently published by Addeo et al. [5, 6, 12, 13]. Including the present series, the cumulative reported experience with RPS for graft reduction comprises only twenty patients, underlining the rarity of this procedure and the limited evidence currently available. To the best of our knowledge, the present study represents the largest pediatric series reported to date and provides the longest follow-up currently available. Although direct comparison between studies is limited by differences in patient selection and surgical indications, the limited available evidence suggests that RPS may provide a rescue option in carefully selected recipients when conventional graft reduction strategies are not feasible.

**Table 5:** Published clinical experience with right posterior sectionectomy as a graft reduction strategy in liver transplantation

Study	Year	Study design	Recipient population	*n*	Surgical approach	Selection criteria	Graft reduction achieved	Major outcomes	Follow-up
Pu et al.	2021	Pilot case series	Adults	5	Ex-vivo RPS	GRWR > 2.5% and GW/RAP > 100 g/cm	Final graft weight 1250–1526 g; final GRWR 2.24–2.44%	No PNF, retransplantation, or mortality	2.1–14.2 months
Rossignol et al.	2023	Case series	Adolescents	3	Ex-situ RPS	GRWR, GW/RAP, and CT volumetry	≈ 20% graft reduction; final GRWR < 4%	One biliary stricture; no PNF or vascular complications	Median 6 months
Zhang et al.	2024	Case report	Adult	1	In-situ RPS	GRWR, GW/RAP, and 3D planning	Graft weight 1603→867 g; GRWR 3.8%→2.0%	IVC stenosis treated by angioplasty; good graft function	12 months
Addeo et al.	2025	Case series	Small-body-size adults	4	Ex-situ RPS	Recipient RAP assessment and liver weight/RAP ratio (> 100 g/cm)	Median graft weight 1654→1365 g; GW/RAP 114→93.3 g/cm	No LFS, retransplantation, vascular or biliary complications; all patients alive	Median 8.8 months (range 6.7–22.2)
Present study	2026	Retrospective case series	Pediatric/adolescent	7	Ex-vivo RPS	Integrated surgical assessment (GRWR, CT anatomy, graft morphology, graft fit)	Median GRWR 4.7%→2.2%; median graft reduction 50%	One primary graft failure requiring retransplantation; one death; six patients alive at last follow-up, including one with a functioning second graft	Median 59.5 months (range 36–82)

*CT* computed tomography, *GRWR* graft-to-recipient weight ratio, *GW/RAP* graft weight-to-recipient right anteroposterior distance ratio, *IVC* inferior vena cava, *PNF* primary non-function, *RPS* right posterior sectionectomy

Overall, our findings are broadly consistent with the limited published experience summarized above. In our series, the observed reduction of GRWR from 4.7% to 2.2% aligns with current recommendations indicating that values above 4% increase the risk of LFS, whereas values below 3% are generally associated with safer outcomes in children [3, 4]. No recipient developed severe LFS with hemodynamic, ventilatory, or graft perfusion compromise. Nevertheless, the occurrence of primary graft failure requiring retransplantation and one death, together with the limited cohort size, precludes firm conclusions regarding the safety of the procedure. In the patient requiring retransplantation, histopathological examination demonstrated extensive ischemic necrosis. No definitive technical cause related to the graft reduction procedure was identified; however, a contribution of technical factors cannot be completely excluded. Therefore, these findings should be interpreted with appropriate caution. Larger multicenter studies are required to better define the safety profile of this technique. Regarding operative efficiency, the reported reduction times should be interpreted as descriptive parameters of the complete back-table procedure rather than as measures of the technical duration of parenchymal transection alone. As such, they also reflect procedural steps inherent to graft preparation before implantation.

An important finding of the present study is that all donor livers included in this cohort were unsuitable for classical split-liver transplantation. Six of the seven grafts violated at least one Eurotransplant criterion for split eligibility, most commonly due to cardiac arrest with prolonged cardiopulmonary resuscitation, excessive cold ischemia time, insufficient donor body weight, advanced donor age, or prolonged ICU stay. The only donor fulfilling all Eurotransplant criteria (Donor 2) presented a high-grade parenchymal injury that was considered to preclude safe splitting. Therefore, in this series, ex-vivo RPS was selected as a rescue option because conventional graft splitting was considered unsuitable based on donor characteristics or intraoperative findings. This contextualizes the use of ex-vivo RPS in situations where implantation of the whole graft in the intended recipient is not feasible and conventional splitting is contraindicated or technically unsuitable. Accordingly, ex-vivo RPS should not be regarded as an alternative to established split-liver techniques, but rather as a highly selected rescue procedure when conventional graft matching is not feasible. The predominance of non-splittable grafts underscores the clinical relevance of reduction techniques in pediatric transplantation, particularly in situations of high urgency, where the opportunity to wait for a size-appropriate graft is limited or absent.

Beyond its technical value, RPS may contribute to a more rational utilization of the donor pool by converting grafts otherwise unsuitable for splitting into viable options for small recipients. It may be particularly valuable in children who are too large for a left lateral segment graft but too small to safely receive a full right lobe. The scarcity of published experience may reflect the rarity of indications, the heterogeneity of surgical expertise, and a general hesitation to perform bench reductions perceived as technically demanding or ischemia-prolonging. A multicenter survey would be valuable to determine how often this strategy is actually used and to collect cumulative experience across centers. Such collaborative efforts could help establish shared selection criteria and better delineate the role of RPS within the spectrum of graft size-reduction strategies in pediatric liver transplantation.

## Conclusion

In highly selected rescue situations, ex-vivo RPS appears to be a technically feasible graft reduction strategy that may facilitate transplantation of oversized whole liver grafts when conventional graft reduction techniques are not feasible. In our experience, this approach enabled substantial graft size reduction while preserving sufficient graft volume, thereby facilitating the use of selected donor grafts that would otherwise have been challenging to transplant in small recipients. However, given the limited sample size and the occurrence of primary graft failure requiring retransplantation and one death within this series, these findings should be interpreted with appropriate caution. Ex-vivo RPS should therefore be regarded as a complementary rescue strategy rather than an alternative to established split-liver techniques. Larger multicenter studies and collaborative registries are required to better define its indications, reproducibility, safety profile, and long-term outcomes.

## Data Availability

The data that support the findings of this study are not publicly available due to privacy and ethical restrictions but are available from the corresponding author upon reasonable request.
